# Adverse childhood experiences, brain efficiency, and the development of pain symptoms in youth

**DOI:** 10.1002/ejp.4702

**Published:** 2024-07-16

**Authors:** Samantha Miller, Karen L. Cobos, Nivez Rasic, Xiangyu Long, Catherine Lebel, Neta Bar Am, Melanie Noel, Daniel Kopala‐Sibley, Richelle Mychasiuk, Jillian Vinall Miller

**Affiliations:** ^1^ Department of Anesthesiology, Perioperative, and Pain Medicine University of Calgary Calgary Alberta Canada; ^2^ Alberta Children's Hospital Research Institute Calgary Alberta Canada; ^3^ Department of Radiology University of Calgary Calgary Alberta Canada; ^4^ Hotchkiss Brain Institute Calgary Alberta Canada; ^5^ Owerko Centre, Alberta Children's Hospital Research Institute Calgary Alberta Canada; ^6^ The Mathison Centre for Mental Health and Education Hotchkiss Brain Institute Calgary Alberta Canada; ^7^ Department of Psychology University of Calgary Calgary Alberta Canada; ^8^ Department of Psychiatry University of Calgary Calgary Alberta Canada; ^9^ Department of Neuroscience Monash University Melbourne Victoria Australia; ^10^ O'Brien Center University of Calgary Calgary Alberta Canada

## Abstract

**Background:**

Adverse childhood experiences (ACEs) are often reported by youths with chronic pain, and both ACEs and chronic pain disrupt how information is processed. However, it is unknown whether changes to shared neural networks underlie the relationship between ACEs and the development of pain symptoms. This study explored the relationships between ACEs, brain efficiency, and pain symptomology in youth.

**Methods:**

A community sample of youths aged 14–18 years underwent MRIs, answered trauma and pain questionnaires, and underwent pain sensory testing, twice, 3 months apart (*N*_*baseline*_ = 44; *N*_*follow‐up*_ = 42). Sensory testing determined thresholds for mechanical and thermal stimuli. Global and local network efficiencies were evaluated using graph theory. Generalized estimating equations were applied to examine whether brain efficiency moderated the relationships between ACEs, pain intensity, and pain sensitivity (i.e., mechanical detection, heat pain, and temperature change thresholds).

**Results:**

There was a significant interaction between ACEs and global brain efficiency in association with pain intensity (*β* = −0.31, *p* = 0.02) and heat pain (*β* = −0.29, *p* = 0.004). Lower global brain efficiency exacerbated the relationship between ACEs and pain intensity (θ_X → Y|W = −1.16_ = 0.37, *p* = 0.05), and heat pain sensitivity (θ_X → Y|W = −1.30_ = 0.44, *p* = 0.05). Higher global brain efficiency ameliorated the relationship between ACEs and pain intensity (θ_X → Y|W = 1.75_ = −0.53, *p* = 0.05).

**Conclusions:**

The relationship between ACEs and pain symptomology was comparable to chronic pain phenotypes (i.e., higher pain intensity and pain thresholds) and may vary as a function of brain efficiency in youth. This stresses the importance of assessing for pain symptoms in trauma‐exposed youth, as earlier identification and intervention are critical in preventing the chronification of pain.

**Significance:**

This article explores the relationship between ACEs, pain symptomology, and brain efficiency in youth. ACEs may affect how the brain processes information, including pain. Youths with lower brain efficiencies that were exposed to more ACEs have pain symptomology comparable to youths with chronic pain. Understanding this relationship is important for the earlier identification of pain symptoms, particularly in vulnerable populations such as youths exposed to trauma, and is critical for preventing the chronification of pain.

## 1 INTRODUCTION

Almost 50% of youths experience at least one adverse childhood experience (ACE) in their lifetime (Groenewald et al., 2020). ACEs are stressful or traumatic events occurring before age 18 and can be categorized as abuse, neglect, or household dysfunction (Herzog & Schmahl, 2018). ACEs hold dose‐dependent effects on the development of physical and psychiatric conditions, including chronic pain (pain ≥3 months), whereby cumulative exposure to ACEs increases one's likelihood of developing persistent pain symptoms (Groenewald et al., 2020). Youths with chronic pain also report experiencing more ACEs (≥1) than their peers (Greene et al., 1985).

ACEs are linked to several risk factors identified in the biopsychosocial model of pain (Kerker et al., 2015; Nelson et al., 2017). The neurobiological processes activated following ACEs overlap with processes involved with altered pain processing in adults with chronic pain (Nelson et al., 2017). ACEs are also linked to post‐traumatic stress symptoms (PTSS; Brockie et al., 2015). PTSS are the prolonged response to trauma and include re‐experiencing, avoidance behaviours, altered cognition, and hyperarousal (American Psychiatric Association [APA], 2013; Brockie et al., 2015). Elevated PTSS are implicated in the chronification of pain and cognitive distortions that influence the appraisal of pain (Nelson et al., 2017; Noel et al., 2016).

Disruptions in functional connectivity are involved in both PTSS and pain symptomology (Sheynin et al., 2020). Resting‐state functional magnetic resonance imaging (rs‐fMRI) studies explore brain functional connectivity, or temporal correlations in BOLD signals in different anatomical regions, which can be interpreted as them being functionally connected (Seitzman et al., 2019). Functional connectivity can be further characterized by how information is communicated across neural networks. Global efficiency measures long‐range information transfer within the entire network (i.e., one region to all other regions of that network), whilst local efficiency assesses information exchange over shorter distances (i.e., adjacent brain regions; Latora & Marchiori, 2001). Adolescents exposed to ACEs have lower global and local brain efficiencies due to poorer information processing and distress (Sheynin et al., 2020). Moreover, lower global efficiency has been indirectly associated with more intense appraisals of noxious stimuli (Zheng et al., 2020). As such, disruptions in brain efficiencies following ACEs may have a critical role in the development of pain symptomology.

Research on the association between ACEs, brain efficiency, and pain in youths is scarce, as it has previously focused on adults. Adolescents are developmentally different and are especially vulnerable to long‐term psychological and somatic outcomes following ACEs (Bremne & Vermetten, 2001). Alterations to brain function during this stage of life can become biologically embedded, which may impede normal brain development. As such, understanding the relationship between neurobiology, ACEs, and pain symptoms in youths is critical, as they may differ from the relationships previously described in adults (Salberg et al., 2022).

This study explored how brain efficiency moderates the relationship between ACEs and pain symptomology in adolescents. We hypothesized that higher exposure to ACEs would be linked to greater pain symptomology in youths with lower global and local brain efficiencies.

## 2 METHODS

This study was approved by the University of Calgary's Conjoint Health Research Ethics Board (REB20‐0078) and conducted in accordance with the Declaration of Helsinki. Written informed consent was obtained from each participant.

### 2.1 Participants and study design

A community sample of youths aged 14–18 years was recruited between October 2020 and February 2022 through online advertisements posted on Kijiji.ca, Facebook, the Child and Adolescent Imaging Research website, and community bulletin boards. Phone interviews were conducted with youths and their guardians who were interested in participating in the study to discuss eligibility and informed consent. Youths were excluded if they had been previously diagnosed with a neurodevelopmental disorder, did not speak English fluently, and/or if they had a contraindication for MRI (e.g., braces). Youths with chronic pain were not excluded from this study as we explored the prevalence of chronic pain in a community sample self‐presenting for a study about trauma and pain. Youths who were eligible and interested in participating in this prospective cohort study booked two lab visits, 3 months apart, at the Alberta Children's Hospital. A 3‐month follow‐up period was chosen for this study as this is the length of time for which recurrent and/or persistent pain is considered chronic (Raffaeli & Arnaudo, 2017). Each lab visit lasted ~ 2 h and the same protocol was used at baseline and follow‐up lab visits to observe if there were any changes in symptomology over time.

**FIGURE 1:**
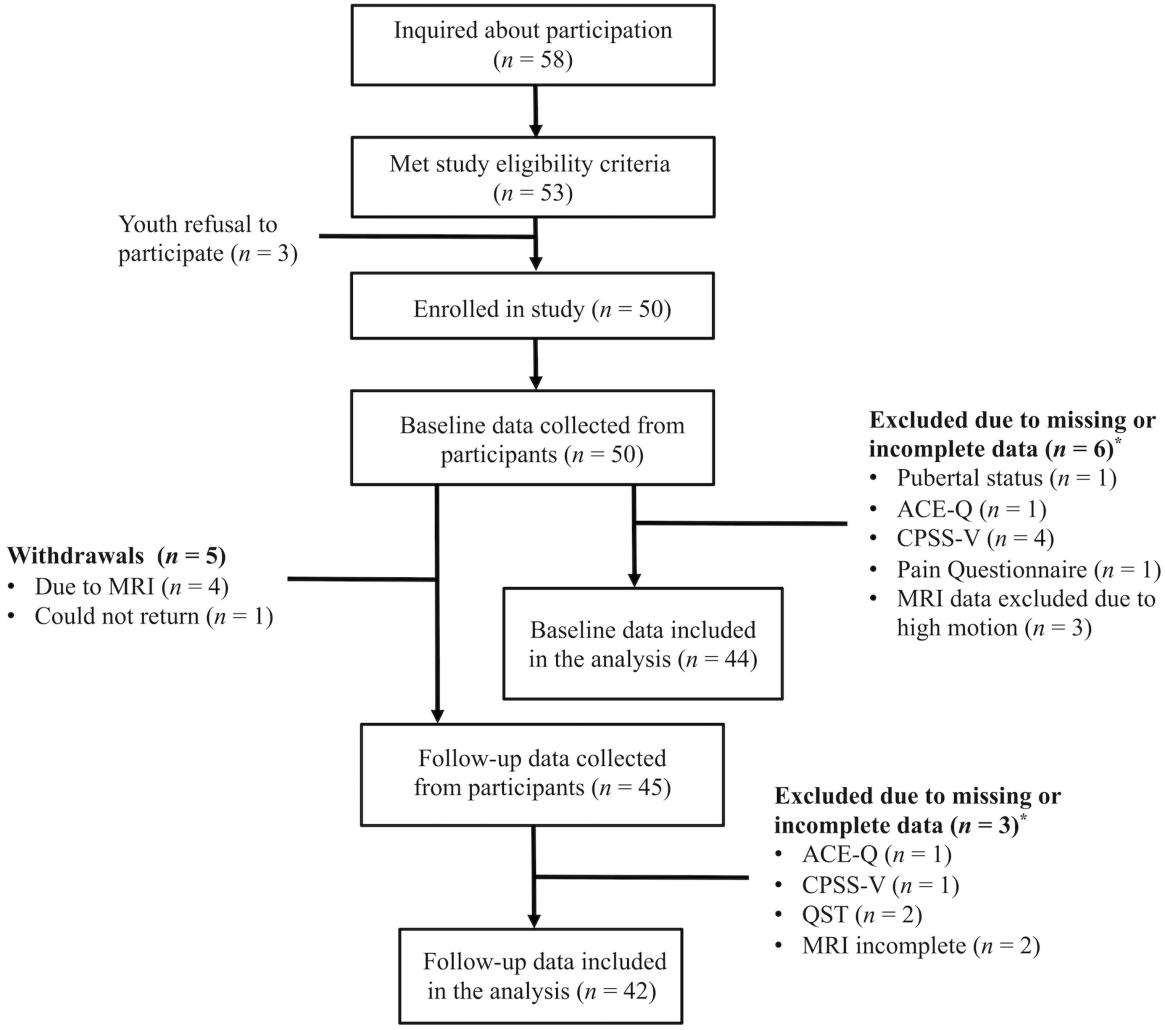
Participant Flow Chart. The flow chart shows the progression of participants in the study. ACE‐Q, Adverse Childhood Experiences Questionnaire; CPSS‐V, The Child PTSD Symptom Scale for DSM‐V; MRI, Magnetic resonance imaging; QST, Quantitative sensory testing. *Participants were removed if missing data in at least one variable of interest. Excluded participants may be missing data in more than one variable.

A participant flowchart is depicted in Figure 1. Of the 58 who inquired about participating, 53 youths met the eligibility criteria. Three of those youths declined to participate. Overall, there were 50 participants enrolled in the study. We obtained baseline data from all 50 youths, and follow‐up data from 45 youths, 3 months later. Five participants withdrew from the study between baseline and follow‐up. Only participants with complete datasets at baseline (*N* = 44) and follow‐up (*N* = 42) were included in this analysis. Little's MCR test determined that youths who did not complete the study were not statistically different from youths who did in terms of pain symptoms (i.e., self‐reported intensity, mechanical detection, cold and warm detection, and heat pain sensitivity; *χ* ^2^ = 6.29, *p* = 0.18) and brain efficiency (*χ* ^2^ = 2.76, *p* = 0.60).

### 2.2 Questionnaires

#### 2.2.1 Demographics

Participants completed a demographic questionnaire, which included questions about their age, sex, gender identity, and ethnicity. Age and gender were used as covariates in the analysis.

#### 2.2.2 Pubertal status

The Self‐Administered Rating Scale for Pubertal Development was used to assess the pubertal status of the participants (Carskadon & Acebo, 1993). Pubertal status was computed from an average rating of five items (range: 1 = ‘not yet started’ to 4 = ‘seems complete’). A validation study found that mean self‐rated development was highly correlated with physician‐rated development (*t* = 0.87–0.84, *p* < 0.01; Carskadon & Acebo, 1993). Pubertal status was included as a covariate in the analysis. Cronbach alpha for the five items was 0.65 at baseline and 0.60 at follow‐up.

#### 2.2.3 Anxiety and depressive symptoms

The National Institutes of Health developed the Patient‐Reported Outcomes Measurement Information System (PROMIS) Paediatric Profile instruments, which are a collection of short forms containing a fixed number of items. Anxiety and depressive symptoms were measured by PROMIS Paediatric Short Forms (8‐item) for youths (Varni et al., 2014). Each item was assessed on a 5‐point Likert scale ranging from 0 (‘never’) to 4 (‘almost always’) using a reporting time frame of the past 7 days. Total raw scores were transformed into standardized t‐scores for analysis. Cronbach alpha for the anxiety and depression short forms were 0.92 and 0.96 at baseline and 0.93 and 0.95 at follow‐up.

#### 2.2.4 Adverse childhood experiences

The Centre for Youth Wellness Adverse Childhood Experiences Questionnaire (ACE‐Q) was administered to calculate the cumulative exposure to ACEs in youths (Burke Harris & Renschler, 2015). Participants only reported on how many of the experiences listed applied to them. The ACE‐Q consists of two sections. The first section includes the 10 traditional ACEs for which there exists population‐level data for disease risk in adults (Anda et al., 2009). The second section includes a 9‐item assessment of exposure to additional early life stressors that have been identified by experts (Burke Harris & Renschler, 2015). The total number of ACEs was calculated for each participant by summing the number of self‐reported ACEs from both sections; the possible range was 0–19. Cronbach alpha for the two items (i.e., number given on each section) was 0.55 at baseline and 0.73 at follow‐up.

#### 2.2.5 Post‐traumatic stress symptoms

PTSS were assessed using the Child PTSD Symptom Scale (CPSS‐V), a 20‐item measure that assesses PTSD symptoms experienced by youths in the past month based on the *Diagnostic and Statistical Manual of Mental Health Disorders* (5th ed.; DSM‐5; APA, 2013) PTSD criteria (Foa et al., 2001). To assess PTSS related to their most traumatic event, participants were asked to reflect on a scary or upsetting thing that bothered them the most to think about, and to keep it in mind whilst completing the questionnaire. The CPSS‐V uses a 5‐point Likert scale, ranging from ‘not at all’ to ‘6 or more times a week/almost always.’ Total symptom severity scores were obtained by summing the 20 items (range: 0–80) with a score of ≥31 indicating clinically elevated PTSD symptoms. The CPSS‐V has excellent internal consistency (*α* = 0.89), good test–retest reliability (*κ* = 0.84), and good convergent validity (*r* = 0.80, *p* < 0.01) with the Child Post‐Traumatic Stress Disorder Reaction Index (Foa et al., 2001). Cronbach alpha for the 20 items was 0.95 at baseline and 0.97 at follow‐up.

#### 2.2.6 Pain presence and intensity

The Pain Questionnaire was used to measure the presence and characteristics of pain (Cornelissen et al., 2014). Youths reported whether they had experienced pain for at least 3 months using a single yes/no item and used an 11‐point scale (0 = ‘no pain’ to 10 = ‘worst pain possible’) to report the average intensity of their pain in the last week. In adolescents, numerical ratings of pain intensity have demonstrated strong convergent validity (*r* = 0.89–0.96) with other validated pain rating scales, such as the Faces Pain Scale‐Revised (Tsze et al., 2018).

### 2.3 Quantitative sensory testing

Quantitative sensory testing (QST) was used to evaluate sensitization through the application of a standardized noxious stimulus. QST objectively establishes thresholds for mechanical detection, cool and warm temperature changes, and heat pain.

Mechanical detection thresholds were assessed using Touch‐Test sensory evaluator filaments (force: 0.008–300 g; Jacob et al., 2015). When participants were not looking, filaments were applied perpendicularly to the back of the left hand. Filaments were first applied in decreasing intensity until they could no longer detect the sensation (i.e., subthreshold). The reverse procedure was then administered whereby the stimulus intensity increased until participants indicated sensation (i.e., suprathreshold). The procedure was repeated until five subthreshold and five suprathreshold values were obtained, and the average filament weight was calculated.

Thermal sensitivity thresholds were determined using the Q‐sense system (Medoc, Israel). A computer‐operated thermode was placed on the back of the left hand and secured by a Velcro strap. The baseline temperature of the Q‐sense system was 32°C and the cut‐off temperatures were 0°C and 50°C. For all tests, the temperature changed at a rate of 1°C/s, and a total of four trials were performed for each threshold: one familiarization trial, followed by three test trials (Jacob et al., 2015). During the warm detection tests, the temperature of the thermode gradually increased and participants pressed a button to indicate when they first felt sensations of warmth. The cool detection tests were set up similarly with the thermode gradually cooling, and participants pressed a button when they began to sense it cool. To determine heat pain thresholds, the thermode gradually became hotter and participants pressed a button when they first felt pain or discomfort. The device returned to baseline temperature at a rate of 1°C/s between the change detection trials and cooled at a rate of 10°C/s for the heat pain trials (Jacob et al., 2015). The temperature of each test trial was recorded, and the mean detection and pain threshold temperatures were determined by averaging the temperatures of the test trials, not including the familiarization trial.

### 2.4 Neuroimaging acquisition

Imaging data were collected using a GE 3 T MR750w (General Electric, Waukesha, WI) scanner with a 32‐channel head coil at the Alberta Children's Hospital, in Calgary. T1‐weighted images were acquired using an FSPGR BRAVO sequence (flip angle = 10°, 226 slices, TR = 8.2 ms, TE = 3.2 ms, voxel size = 0.8 × 0.8 × 0.8 mm, matrix size = 512 × 512, inversion time = 600 ms, scan duration = 5:31 min). Resting‐state images were obtained using a gradient‐echo‐planar imaging sequence (TR = 2 s, TE = 30 ms, flip angle = 60°, 36 slices, voxel size = 3.6 × 3.6 × 3.6 mm, matrix size = 64 × 64, 240 vol, scan duration = 8:10 min) whilst participants watched an 8‐minute clip from a National Geographic film.

#### 2.4.1 Image preprocessing

MRI data preprocessing was completed in AFNI and FSL (Cox, 1996; Jenkinson et al., 2012). For each participant, the T1‐weighted images were skull‐stripped and segmented into grey matter, white matter, and cerebral spinal fluid (CSF) image masks that were then co‐registered to their fMRI space. The data were corrected for slice timing and head motion. A 36‐parameter matrix was created by averaging the signals from the whole‐brain mask, CSF mask, white matter mask, six head motion parameters, and their temporal derivatives and quadratic term signals (Satterthwaite et al., 2013). A spike matrix was then created from volumes that had high relative frame‐wise displacement (FD, >0.3 mm) (Power et al., 2014). Afterwards, the 36‐parameter matrix and the spike matrix were regressed out of the fMRI signals. If a participant's dataset had spike volumes longer than 4 min (50% of overall scan time), they were excluded from the analysis (Satterthwaite et al., 2013). We removed three participants from our analysis due to high head motion at baseline (i.e., less than 4 minutes of fMRI data with FD <0.3 mm). Finally, the remaining fMRI signals were band‐pass filtered (0.009–0.08 Hz), underwent linear trend removal, spatially transformed to the Montreal Neurological Institute (MNI) standard space using an adolescent template ages 13–18.5 years old, and were spatially smoothed using a 4 mm full width at half maximum kernel (Fonov et al., 2011).

The slice timing, head motion correction, image segmentation, head motion detection, co‐registration, and spatial normalization and smoothing were performed in FSL, version FSL_6.0.5 (Jenkinson et al., 2012). The regression of the nuisance signals, band‐pass filtering, and linear trend removal were completed in AFNI, version AFNI_21.3.13 (Cox, 1996).

#### 2.4.2 Functional connectome construction

The Automatic Anatomical Labeling (AAL) template was used to subdivide each brain into 90 different regions, omitting the cerebellum (Tzourio‐Mazoyer et al., 2002). Within each AAL region, the average time series was computed, and Pearson's correlation coefficients were calculated between the averaged time series for each of the AAL regions and z‐transformed to create a 90 × 90 connectivity matrix.

#### 2.4.3 Graph theory calculations of network efficiency

Graph theoretical measurements were calculated for each thresholded and binarized connectivity matrix within the whole time series (Wozniak et al., 2017). The threshold of the matrix was set at *r* = 0.13 (*p* < 0.05) to remove any non‐significant correlations and pseudo‐connectivities from the connectivity matrix. The GRETNA toolbox computed the whole‐brain graph theory metrics (i.e., global and local efficiency) for each participant (Wang et al., 2015). Results were displayed using the BrainNet Viewer toolbox, version 1.7, on MATLAB (Xia et al., 2013).

### 2.5 Statistical analyses

Analyses were conducted using the Statistical Package for the Social Sciences (SPSS), version 28.0 (IBM Corp., 2021). Data were assessed for violations of normality using the Shapiro–Wilk test. The following variables were found to deviate from normality: age, pubertal status, PTSS, ACEs, pain intensity, and mechanical, warm and cold detection thresholds. List‐wise deletion of missing data was implemented, and analyses were conducted using two‐tailed hypothesis testing.

Demographic characteristics, brain efficiency, and pain symptomology at baseline and follow‐up were examined using paired‐sample *t*‐tests and Chi‐square, where appropriate, to determine if variables significantly changed between lab visits. In cases of non‐normality, the Wilcoxon signed‐rank test was applied. Spearman rank correlations were used to examine the relationships between ACEs, PTSS, anxiety, and depressive symptoms at baseline and follow‐up.

Multivariate analyses were performed using generalized estimating equations (GEEs) to examine relationships with both baseline and follow‐up variables. First, GEEs were used to identify if there was a significant relationship between ACEs and whole‐brain efficiency. Then GEEs were used to explore whether brain efficiency moderated the relationship between ACEs and pain symptomology (intensity and mechanical, cool, warm detection, and heat pain thresholds). GEE models accounted for age, gender, pubertal status, anxiety and depressive symptoms, and PTSS. Corrections for multiple comparisons were applied using the Benjamini‐Hochberg procedure for false discovery rates for all models of global efficiency and local efficiency combined. We then utilized the PROCESS macro in SPSS, which implements the Johnson‐Neyman test to determine the conditional effects of the focal predictor (i.e., ACEs) on the outcome (i.e., pain symptomology) at varying values of the moderator (i.e., brain efficiency; Hayes, 2012). This tests, for example, whether there is a stronger association between ACEs and pain intensity at high vs. low levels of brain efficiency. The GEEs and figures included data from both time points (*N*_*Baseline*_ = 44, *N*_*Follow‐up*_ = 42). Advantages to GEEs are that they can be used to analyse non‐normal data, they use all available data for each subject, and they account for correlations between variables over time within the same individual.

## 3 RESULTS

### 3.1 Sample characteristics

Of the 44 youths with complete data at baseline, the median age was 16 years (IQR: 15–17) and the sample was predominantly white (68%) and female (64%). Forty‐two youths had complete data at follow‐up. Similarly, the median age was 16 years (IQR: 15–17) and the sample was predominantly white (64%) and female (57%). Ten youths reported experiencing pain for ≥3 months at baseline (23%) and follow‐up (24%) with no significant changes in reported chronic pain status between lab visits. (*χ* ^*2*^ = 0.01, *p* = 0.91). As expected with time, the median age (*Z* = 4.00, *p* < 0.01) and pubertal status scores (*Z* = 2.47, *p* = 0.01) significantly changed between baseline and follow‐up. There were no significant changes in pain symptomology or brain efficiency over the 3‐month study period (all *p* > 0.05). Characteristics of the sample are shown in Table 1. ACEs were moderately corrected with PTSS, and anxiety and depressive symptoms (baseline: *ρ* = 0.64, *ρ* = 0.48, *ρ* = 0.41; follow‐up: *ρ* = 0.66, *ρ* = 0.37, *ρ* = 0.47). The predominant themes of the traumatic events identified by youths on the CPSS‐V are listed in Table S1. Of the responses, most youths listed events that align with concerns for the mental health and well‐being of a family member or friend as their most significant trauma at baseline (*N* = 6; 14%) and follow‐up (*N* = 7; 17%). Seven youths at baseline (16%) and nine youths at follow‐up (21%) did not specify a traumatic event. Four youths at baseline (9%) and three youths at follow‐up (7%) reported that they had not experienced anything traumatic.

**TABLE 1:** Cohort characteristics.

Characteristics	Baseline (*n* = 44)	Follow‐up (*n* = 42)	*p*‐value
Age, Median (IQR), year	16.00 (15.00–17.00)	16.00 (15.00–17.00)	<0.01*
Sex (female), *n* (%)	30 (68)	26 (62)	0.54
Gender (female), *n* (%)	28 (64)	24 (57)	0.63
Ethnicity, *n* (%)			0.91
Aboriginal	3 (7)	2 (5)	
Arab/West Asian	1 (2)	1 (2)	
Black	1 (2)	3 (7)	
South Asian	3 (7)	4 (10)	
White/Caucasian	30 (68)	27 (64)	
Multiethnic	6 (14)	5 (12)	
Pubertal Status, Median (IQR)	3.40 (2.80–3.80)	3.41 (3.00–3.80)	0.01*
Anxiety T‐Score	49.7 (11.1)	49.7 (12.0)	0.99
Depression T‐Score, M (SD)	64.8 (12.9)	64.2 (10.9)	0.69
Total PTSS Score, Median (IQR)	14.00 (6.25–42.00)	13.50 (0.00–40.25)	0.24
Total ACEs, Median (IQR)	5.00 (1.25–7.00)	4.00 (2.00–7.25)	0.40
Pain Intensity Rating (1–10), Median (IQR)	3.00 (1.25–5.00)	3.00 (0.00–4.25)	0.47
Mechanical stimuli detection threshold (g), Median (IQR)	0.11 (0.06–0.30)	0.11 (0.04–0.29)	0.10
Warm detection threshold (°C), Median (IQR)	33.73 (33.42–34.57)	33.92 (33.31–35.52)	0.61
Cold detection threshold (°C), Median (IQR)	31.05 (30.53–31.33)	30.98 (30.06–31.41)	0.99
Heat pain threshold (°C), M (SD)	42.10 (3.86)	42.71 (3.96)	0.20
Pain for ≥3 months, *n* (%)	10 (23)	10 (24)	0.91
Global Efficiency, M (SD)	0.27 (0.01)	0.27 (0.01)	0.33
Local Efficiency, M (SD)	0.35 (0.03)	0.34 (0.02)	0.32

*p ≤ 0.05.

### 3.2 Whole‐brain efficiency and ACEs

The relationship between ACEs and whole‐brain efficiency measures is shown in Table S2. ACEs were inversely associated with global efficiency (*β* = −0.23, *p* = 0.03) and local efficiency (*β* = −0.26, *p* = 0.007) after accounting for age (*p* > 0.05), gender (*p* > 0.05), pubertal status (*p* > 0.05), anxiety (*p* > 0.05), depression (*p* > 0.05), and mean PTSS (*p* > 0.05).

### 3.3 ACEs, brain efficiency, and pain intensity

**FIGURE 2:**
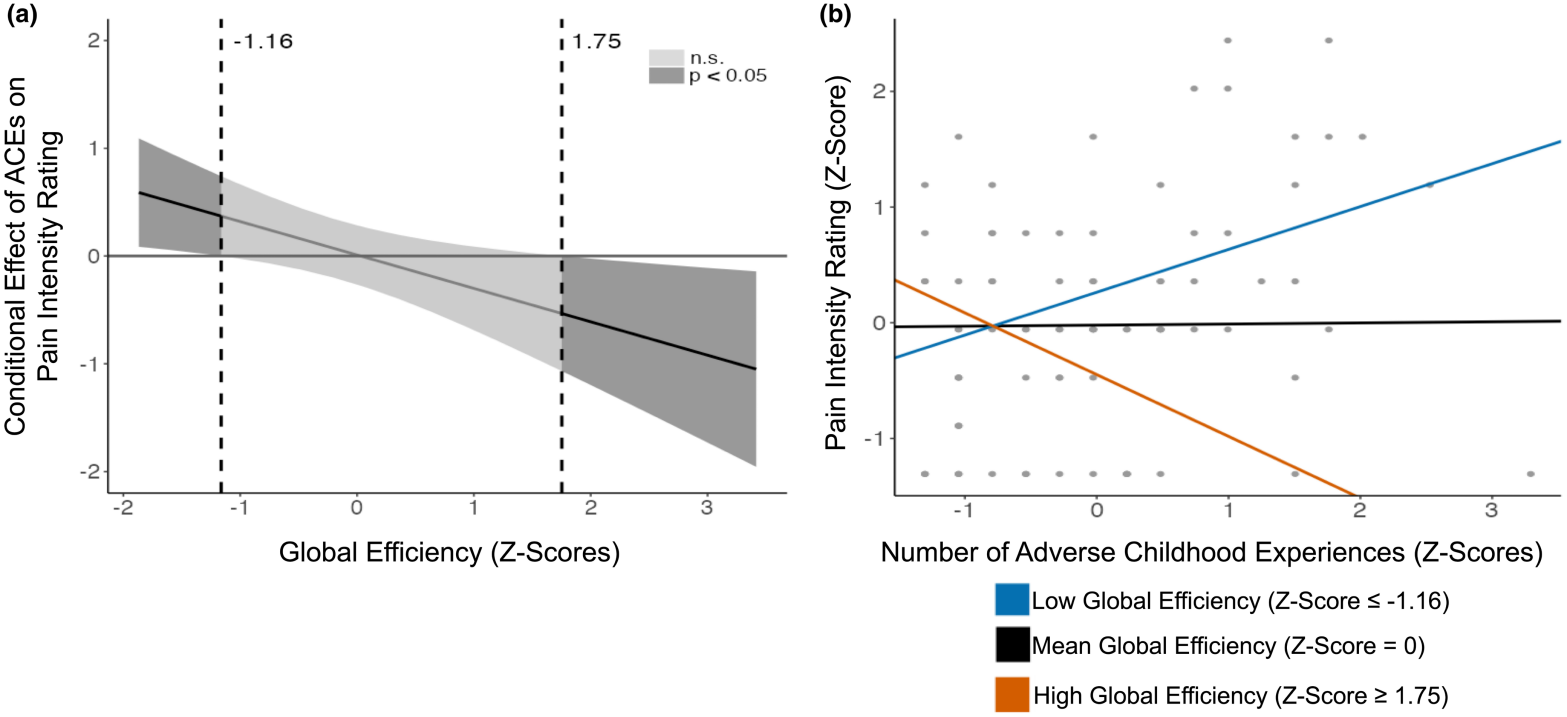
Moderating Effect of Global Brain Efficiency on ACEs and Pain Intensity. (a) Johnson‐Neyman plot and confidence bands for the conditional relationship between ACEs and pain intensity as a function of global brain efficiency. (b) Plot visualizing the conditional effects of global efficiency on the relationship between ACEs and pain intensity ratings. Regression slopes for low (≤0.25; *p* = 0.05), average (0.27; *p* = 0.93), and high (≥0.28; *p* = 0.05) global brain efficiencies were plotted over individual data points. This model explains 28% of the total variance. Included in the analysis were *N*_*Baseline*_ = 44 and *N*_*Follow‐up*_ = 42. Some data points overlap. N.s., non‐significance.

There was a significant interaction between ACEs and global efficiency when predicting average pain intensity in the last week (*β* = −0.31, *p* = 0.02; see Table 2). This relationship remained significant following corrections for multiple comparisons (*p* < 0.02). Johnson‐Neyman analyses revealed that the number of ACEs youths were exposed to were significantly associated with self‐reported pain intensity ratings when brain efficiency was both low (≤ −1.16) and high (≥1.75; see Figure 2a). Regression slopes of ACEs and pain intensity ratings were plotted separately for adolescents with low, average, and high global efficiencies (see Figure 2b). Whilst a significant positive relationship was found between ACEs and pain intensity ratings in youths with low global efficiencies (θ_X → Y|W = ‐1.16_ = 0.37, *p* = 0.05), a significant negative relationship was described in youths with high global efficiencies (θ_X → Y|W = 1.75_ = −0.53, *p* = 0.05). This relationship was not significant for youths with average brain efficiencies (θ_X → Y|W = 0.08_ = −0.01, *p* = 0.91).

**TABLE 2:** The effects of brain efficiency and ACEs on pain intensity.

	Pain intensity					
	Global efficiency			Local efficiency		
	*β*	95% CI	*p*‐value	*β*	95% CI	*p*‐value
Age	0.03	[−0.20, 0.25]	0.83	0.03	[−0.21, 0.27]	0.81
Gender	0.13	[−0.07, 0.34]	0.21	0.13	[−0.08, 0.34]	0.24
Puberty	−0.11	[−0.31, 0.10]	0.30	−0.09	[−0.30, 0.13]	0.44
Anxiety	0.18	[−0.08, 0.44]	0.17	0.18	[−0.09, 0.45]	0.19
Depression	−0.12	[−0.34, 0.10]	0.28	−0.10	[−0.32, 0.13]	0.39
PTSS	0.30	[0.07, 0.53]	0.01*	0.29	[0.04, 0.54]	0.03*
ACEs	0.01	[−0.26, 0.28]	0.95	0.02	[−0.31, 0.35]	0.92
Brain Efficiency	−0.24	[−0.42, −0.07]	0.01*	−0.21	[−0.37, −0.05]	0.01*
Brain Efficiency X ACEs	−0.31	[−0.57, −0.05]	0.02*	−0.17	[−0.35, 0.01]	0.07

Abbreviations: ACEs, number of exposures to adverse childhood experiences; PTSS, mean post‐traumatic stress symptom score.

*p ≤ 0.05.

Local brain efficiency did not significantly moderate the relationship between ACEs and pain intensity ratings in youths (*β* = −0.17, *p* = 0.07; see Table 2).

### 3.4 ACEs, brain efficiency, and sensitivity to mechanical stimuli

Global brain efficiency did not significantly moderate the relationship between ACEs and mechanical detection thresholds in youths (*β* = −0.28, *p* = 0.08; see Table 3).

**TABLE 3:** The effects of global brain efficiency and ACEs on pain sensitivity.

	Mechanical detection threshold			Warm detection threshold		
	*β*	95% CI	*p*‐value	*β*	95% CI	*p*‐value
Age	−0.07	[−0.26, 0.12]	0.47	−0.13	[−0.30, 0.05]	0.15
Gender	0.33	[−0.06, 0.73]	0.10	0.06	[−0.29, 0.42]	0.73
Puberty	−0.30	[−0.58, −0.02]	0.04*	−0.22	[−0.54, 0.10]	0.18
Anxiety	−0.29	[−0.68, 0.09]	0.14	−0.05	[−0.30, 0.20]	0.68
Depression	−0.12	[−0.41, 0.18]	0.44	0.001	[−0.37, 0.37]	1.00
PTSS	0.27	[−0.26, 0.81]	0.32	−0.02	[−0.35, 0.32]	0.91
ACEs	0.08	[−0.40, 0.56]	0.74	−0.02	[−0.19, 0.14]	0.80
Global Efficiency	−0.11	[−0.24, −0.01]	0.08	−0.22	[−0.42, −0.02]	0.03*
Global Efficiency X ACEs	−0.28	[−0.59, 0.03]	0.08	−0.22	[−0.44, −0.01]	0.04*

	Cold detection threshold			Heat pain threshold		
	*β*	95% CI	*p*‐value	*β*	95% CI	*p*‐value
Age	0.16	[0.03, 0.30]	0.02*	−0.05	[−0.29, 0.20]	0.71
Gender	−0.06	[−0.49, 0.37]	0.78	−0.21	[−0.46, 0.04]	0.10
Puberty	0.14	[−0.19, 0.48]	0.41	0.06	[−0.23, 0.34]	0.69
Anxiety	0.04	[−0.24, 0.32]	0.79	0.13	[−0.20, 0.46]	0.45
Depression	0.10	[−0.32, 0.54]	0.63	0.004	[−0.39, 0.40]	0.98
PTSS	−0.16	[−0.42, 0.10]	0.23	−0.06	[−0.29, 0.16]	0.59
ACEs	0.06	[−0.09, 0.20]	0.46	0.07	[−0.18, 0.31]	0.59
Global Efficiency	0.08	[−0.05, 0.20]	0.22	−0.11	[−0.30, 0.07]	0.23
Global Efficiency X ACEs	0.09	[−0.09, 0.26]	0.32	−0.29	[−0.49, −0.09]	0.004*

Abbreviations: ACEs, number of exposures to adverse childhood experiences; PTSS, mean post‐traumatic stress symptom score.

*p ≤ 0.05.

There was a significant interaction between ACEs and local brain efficiency when predicting sensitivity to mechanical stimuli (*β* = −0.29, *p* = 0.04; see Table 4). However, this interaction did not survive corrections for multiple comparisons (*p* > 0.02).

**TABLE 4:** The effects of local brain efficiency and ACEs on pain sensitivity.

	Mechanical detection threshold			Warm detection threshold		
	*β*	95% CI	*p*‐value	*β*	95% CI	*p*‐value
Age	−0.06	[−0.22, 0.11]	0.49	−0.13	[−0.30, 0.05]	0.15
Gender	0.36	[−0.05, 0.76]	0.08	0.07	[−0.28, 0.42]	0.70
Puberty	−0.32	[−0.61, −0.04]	0.03*	−0.22	[−0.54, 0.11]	0.19
Anxiety	−0.31	[−0.68, 0.07]	0.11	−0.06	[−0.29, 0.18]	0.64
Depression	−0.11	[−0.40, 0.17]	0.43	−0.003	[−0.37, 0.36]	0.99
PTSS	0.31	[−0.19, 0.81]	0.22	−0.002	[−0.33, 0.32]	0.99
ACEs	0.04	[−0.39, 0.47]	0.85	−0.03	[−0.20, 0.13]	0.71
Local Efficiency	−0.16	[−0.31, −0.02]	0.03*	−0.18	[−0.37, 0.02]	0.08
Local Efficiency X ACEs	−0.29	[−0.57, −0.01]	0.04*	−0.21	[−0.38, −0.03]	0.02*

	Cold detection threshold			Heat pain threshold		
	*β*	95% CI	*p*‐value	*β*	95% CI	*p*‐value
Age	0.16	[0.04, 0.29]	0.01*	−0.03	[−0.28, 0.21]	0.79
Gender	−0.08	[−0.51, 0.36]	0.74	−0.20	[−0.47, 0.06]	0.13
Puberty	0.15	[−0.18, 0.49]	0.37	0.06	[−0.21, 0.33]	0.67
Anxiety	0.04	[−0.23, 0.32]	0.75	0.13	[−0.21, 0.46]	0.47
Depression	0.12	[−0.31, 0.54]	0.59	0.10	[−0.39, 0.41]	0.96
PTSS	−0.18	[−0.46, 0.09]	0.19	−0.06	[−0.27, 0.16]	0.63
ACEs	0.08	[−0.07, 0.22]	0.31	0.06	[−0.21, 0.34]	0.67
Local Efficiency	0.08	[−0.05, 0.20]	0.23	−0.09	[−0.28, 0.09]	0.33
Local Efficiency X ACEs	0.14	[−0.01, 0.29]	0.06	−0.23	[−0.43, −0.03]	0.03*

Abbreviations: ACEs, number of exposures to adverse childhood experiences; PTSS, mean post‐traumatic stress symptom score.

*p ≤ 0.05.

### 3.5 ACEs, brain efficiency, and sensitivity to warm temperature

There was a significant interaction between ACEs and global brain efficiency when predicting sensitivity to warm temperature changes (*β* = −0.22, *p* = 0.04; see Table 3). However, this interaction did not survive corrections for multiple comparisons (*P* > 0.02).

There was a significant interaction between ACEs and local brain efficiency when predicting sensitivity to warm temperature changes (*β* = −0.21, *p* = 0.02; see Table 4). However, this interaction did not survive corrections for multiple comparisons (*P* > 0.02).

### 3.6 ACEs, brain efficiency, and sensitivity to cold temperature

Neither global brain efficiency (*β* = 0.09, *p* = 0.32; see Table 3), nor local brain efficiency (*β* = 0.14, *p* = 0.06; see Table 4) moderated the relationship between ACEs and cold temperature detection thresholds in youths.

### 3.7 ACEs, brain efficiency, and sensitivity to heat pain

**FIGURE 3:**
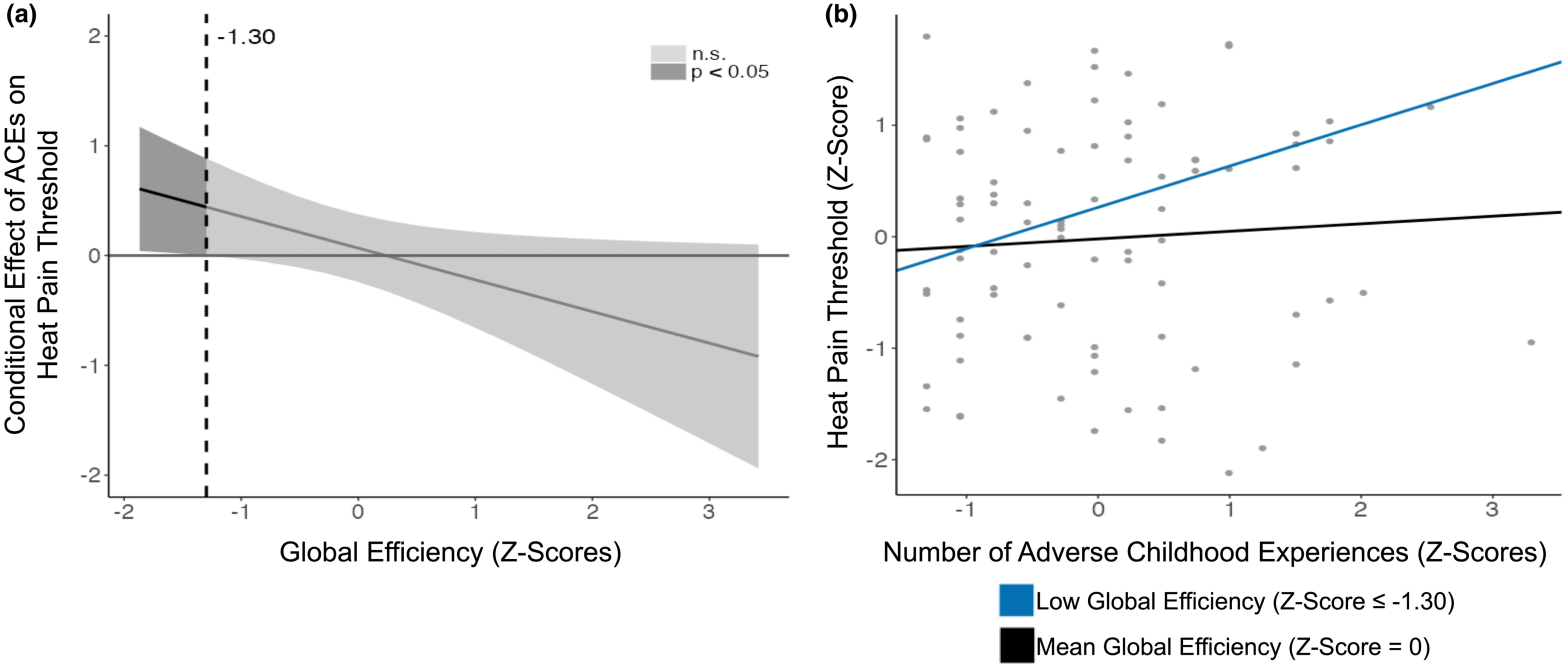
Moderating Effect of Global Brain Efficiency on ACEs and Heat Pain Sensitivity. (a) Johnson‐Neyman plot and confidence bands for the conditional relationship between ACEs and heat pain thresholds as a function of global brain efficiency. (b) Plot visualizing the conditional effects of global efficiency on the relationship between ACEs and heat pain thresholds. Regression slopes for low (≤0.25; *p* = 0.05) and average (0.27; *p* = 0.74) global brain efficiencies were plotted over individual data points. This model explains 10% of the total variance. Included in the analysis were *N*_*Baseline*_ = 44, *N*_*Follow‐up*_ = 42. Some data points overlap. N.s., non‐significance.

There was a significant interaction between ACEs and global brain efficiency when predicting sensitivity to heat pain (*β* = −0.29, *p* = 0.004; see Table 3). This relationship remained significant following corrections for multiple comparisons (*p* < 0.02). Johnson‐Neyman analyses revealed that youths ACEs were significantly associated with heat pain thresholds when brain efficiency was low (≤ −1.30; see Figure 3a). Regression slopes of ACEs and heat pain thresholds were plotted separately for adolescents with low and average global brain efficiencies (see Figure 3b). Whilst a significant postive relationship was found between ACEs and heat pain thresholds in youths with low global efficiencies (θ_X → Y|W = ‐1.30_ = 0.44, *p* = 0.05), this relationship was not significant in youths with average global brain efficiences (θ_X → Y|W = ‐0.02_ = 0.07, *p* = 0.64).

There was a significant interaction between ACEs and local brain efficiency when predicting sensitivity to heat pain (*β* = −0.23, *p* = 0.03; see Table 4). However, this interaction did not survive corrections for multiple comparisons (*p* > 0.02).

## 4 DISCUSSION

This study examined the relationship between ACEs, brain efficiency, and pain symptomology in a community sample of adolescents. Over the 3‐month period, brain efficiency and pain symptomology did not significantly change; however, higher ACEs were associated with lower global and local brain efficiencies. Amongst youths with lower global brain efficiency, greater exposure to ACEs was associated with higher pain intensity ratings and lower heat pain thresholds. In contrast, amongst youths with higher brain efficiency, greater exposure to ACEs was associated with lower pain intensity ratings.

Global brain efficiency can estimate the potential for information integration (Lenoir et al., 2021). Higher global efficiency is associated with an increased capacity to process information and higher cognitive control (Lenoir et al., 2021; Schulte et al., 2020). Local brain efficiency, on the other hand, is associated with information segregation (Lenoir et al., 2021). A highly segregated network consists of many distinct subnetworks may allow faster processing and greater resilience to disturbances (Wang et al., 2021; Wig, 2017). The present study identified that greater exposure to ACEs was significantly related to lower global and local brain efficiencies. To the best of our knowledge, no existing studies have examined the relationship between ACEs and the adolescent brain using a network‐based approach. However, in young adults, exposure to more ACEs is also correlated with lower global brain efficiency (Ohashi et al., 2017). Therefore, it would appear that ACEs influence brain efficiency and related cognitive processes during adolescence through what may be persistent neuroactivation and excitotoxicity (Lowry et al., 2022).

Brain connectivity often changes in response to intrinsic and extrinsic stimuli, a phenomenon known as neuroplasticity (Mateos‐Aparicio & Rodríguez‐Moreno, 2019). Non‐linear developmental trajectories of global brain efficiency during adolescence have been described as a function of age and pubertal status (Gracia‐Tabuenca et al., 2021; Koenis et al., 2018). Studies have reported a decrease in global efficiency in early adolescence (i.e., first signs of puberty), followed by an increase in global efficiency until the end of puberty, and then levelling off during early adulthood. As such, global efficiency may temporarily decrease with large‐scale topical reorganization but ultimately stabilize over time. In the present study, we observed a decrease in global efficiency in response to exposure to more ACEs, even after controlling for age and pubertal status. This decrease in global efficiency is the opposite of what is normally observed during adolescence. Exposure to more ACEs may induce changes to functional networks that alter cognitive processing (Berken et al., 2021).

The relationship between ACEs and pain symptomology appears to vary as a function of global brain efficiency. Specifically, lower global brain efficiency significantly amplified the relationship between ACEs and pain intensity (more intense pain) and heat pain sensitivity (higher pain thresholds). Several cognitive processes are implicated in the perception and evaluation of painful stimuli, whereby deficits in those processes are commonly reported by patients with chronic pain (Khera & Rangasamy, 2021). In pain populations, the relationship between global brain efficiency and pain intensity has not been explored. However, it has been theorized that patients with chronic pain report more intense pain because of poor information processing (Reckziegel et al., 2019). Further, lower brain efficiency is associated with decreased cognitive control, which is critical for the downregulation of pain signals (Khera & Rangasamy, 2021; Schulte et al., 2020). Individuals with lower brain efficiency (i.e., poor cognitive control) may be less able to inhibit pain signals and therefore report more intense pain (Khera & Rangasamy, 2021). In contrast, better cognitive control may be important in mitigating the effects of ACEs on pain symptomology by improving one's ability to integrate information and downregulate pain signals. However, this should be further investigated as the associations between higher global efficiency and lower pain intensity may be a maladaptive response to trauma (Korem et al., 2022; Wheelock et al., 2018).

Subjective reports of pain may not fully characterize painful experiences. Therefore, standardized measures, like QST, may offer unique insights into the mechanisms underlying pain symptoms (Weaver et al., 2021). We identified that global efficiency moderated the association between ACEs and heat pain sensitivity. However, ACEs and sensitivity to mechanical and warm and cold thermal stimuli changes did not vary as a function of brain efficiency. These findings may be partially explained by studies suggesting that upper‐body sites (i.e., hands) are more sensitive to thermal stimuli than mechanical stimuli (Hagander et al., 2000; Neziri et al., 2011). Other studies have found differences in thermal pain and detection thresholds (Malmström et al., 2015). Therefore, future work should consider additional body sites for conducting QST, and longer‐term follow‐up to better characterize the relationships between ACEs, brain efficiency, and pain thresholds.

Exposure to childhood adversity is associated with maladaptive neurobiological changes, including central sensitization (Müller, 2000). Central sensitization is implicated in the chronification of pain, whereby enhanced communication in pain processing networks can lead to pain hypersensitivity (Müller, 2000). Central sensitization manifests as increased sensitivity to heat pain during the chronification of back pain in adults (Granovsky & Yarnitsky, 2013). However, adolescents with concurrent pain and PTSS experience the opposite effect. Youths with chronic pain and elevated PTSS were less sensitive to thermal pain during the cold‐water pressor task (Janssen et al., 2022). These findings suggest that pain sensitization may be different for youths than adults and that trauma can alter pain sensitivity. Adolescents exposed to more ACEs may have higher pain thresholds that vary as a function of global brain efficiency.

PTSS was not associated with brain efficiency in this study. Although lower global efficiency is related to exposure to ACEs in adolescents, it has not been linked to PTSS (Cisler et al., 2018; Suo et al., 2015; Xu et al., 2018). In adults, associations between PTSS and brain efficiency have been reported (Lei et al., 2015). Our results are consistent with the existing literature and suggest that ACEs are more important than PTSS regarding brain efficiency in youth. Moreover, these results further support that youths may be more vulnerable to the effects of trauma, given that not everyone exposed to ACEs will develop PTSS (Brockie et al., 2015).

This study has several limitations. Additional factors such as the type of ACEs experienced, the frequency and length of exposure, and when the ACEs occurred may influence brain functional connectivity and pain outcomes in youths (Cassiers et al., 2018; Graf et al., 2021; Larsen & Luna, 2018; Nelson et al., 2020; Tidmarsh et al., 2022). Future research should examine whether the type and timing of ACEs are associated with the chronification of pain in youths with lower brain efficiency. Further, in the present study, 10 youths self‐reported persistent pain at both lab visits. Importantly, this is reflective of the population rates of chronic pain (King et al., 2011; Stanford et al., 2008). We could not isolate these youths in our study due to our small sample size, therefore their inclusion may have confounded the findings. Results from this study will be used to inform power analyses for future studies following trauma‐exposed youths with and without chronic pain at baseline to compare their pain trajectories. Moreover, other tools will be considered for assessing pubertal status and ACEs given that the internal consistencies of the Self‐Administered Rating Scale for Pubertal Development and ACE‐Q were relatively low, and it is unclear how they may have impacted our ability to detect relationships with these variables. In particular, the utility of the ACE‐Q is limited for research purposes, as is not a validated diagnostic tool, and it was developed for the sole purpose of information gathering/sharing (Burke Harris & Renschler, 2015).

It has been long hypothesized that the relationship between exposure to ACEs and the chronification of pain varies with brain function (Holley et al., 2016; Vinall et al., 2016). We demonstrated that global brain efficiency moderates the relationship between ACEs and pain symptomology in youth. Lower global efficiency (≤0.25) reinforced the relationships between ACEs, pain intensity, and sensitivity to heat pain. This may be explained by poor information processing during pain appraisal, thereby leading to higher reported pain intensity and higher thresholds for heat painful stimuli (Khera & Rangasamy, 2021; Wolff et al., 2016). More intense pain ratings and higher pain thresholds are reflective of chronic pain phenotypes. Therefore, brain efficiency may be critical in the chronification of pain for youths exposed to ACEs. However, more research is needed to understand how important brain efficiency is in this relationship. These findings stress the importance of assessing for changes in pain symptoms in trauma‐exposed youths as they may be at a greater risk of developing chronic pain. The transition from acute to chronic pain is a critical period for intervention implementation. Earlier identification of trauma and pain symptomology may be important in reducing the burden of chronic pain (Reckziegel et al., 2019).

## AUTHOR CONTRIBUTIONS

**Samantha Miller:** Software, Validation, Formal analysis, Investigation, Data curation, Writing – Original draft, Writing – Review and editing, Visualization. **Karen L. Cobos:** Software, Validation, Writing – Review and editing, Supervision. **Nivez Rasic:** Resources, Writing – Review and editing. **Xiangyu Long:** Methodology, Software, Validation, Resources, Writing – Review and editing, Supervision. **Catherine Lebel:** Methodology, Validation, Resources, Writing – Review and editing. **Neta Bar Am:** Investigation, Data curation, Writing – Review and editing, Project administration. **Melanie Noel:** Conceptualization, Resources, Writing – Review and editing. **Daniel Kopala‐Sibley:** Validation, Writing – Review and editing. **Richelle Mychasiuk:** Conceptualization, Writing – Review and editing. **Jillian Vinall Miller:** Conceptualization, Methodology, Formal analysis, Investigation, Resources, Data curation, Writing – Review and editing, Visualization, Investigation, Project administration, Funding acquisition. All authors discussed the results and contributed to critically revising the article. All authors read and approved the final version of the article.

## FUNDING INFORMATION

This research was supported by an Early Career Investigator Pain Research Grant from the Canadian Pain Society/Pfizer Canada to Dr. Miller (10030228). Further support was received by generous community donations to the Vi Riddell Pain & Rehabilitation Centre through the Alberta Children's Hospital Foundation (10,016,199 to Dr. Rasic) and (10,027,696 to Dr. Miller). Start‐up funding was also provided by the Alberta Children's Hospital Research Institute (10021622) to Dr. Miller. Samantha Miller is supported by a NSERC Canada Graduate Scholarship Master's scholarship.

## CONFLICT OF INTEREST STATEMENT

The authors report no conflicts of interest.

## Supporting information


Tables S1–S2.

## References

[ejp4702-bib-0001] American Psychiatric Association . (2013). Diagnostic and statistical manual of mental disorders (5th ed.). American Psychiatric Association. 10.1176/appi.books.9780890425596

[ejp4702-bib-0002] Anda, R. F. , Dong, M. , Brown, D. W. , Felitti, V. J. , Giles, W. H. , Perry, G. S. , Valerie, E. J. , & Dube, S. R. (2009). The relationship of adverse childhood experiences to a history of premature death of family members. BMC Public Health, 9, 106. 19371414 10.1186/1471-2458-9-106 PMC2674602

[ejp4702-bib-0003] Berken, J. A. , Heard‐Garris, N. , & Wakschlag, L. S. (2021). Guardians at the gate: Early adversity, neurocognitive development, and the role of the pediatrician in the era of COVID‐19. Frontiers in Pediatrics, 9, 665335. 10.3389/fped.2021.665335 33937157 PMC8079717

[ejp4702-bib-0004] Bremne, J. D. , & Vermetten, E. (2001). Stress and development: Behavioral and biological consequences. Development and Psychopathology, 13(3), 473–489. 10.1017/s0954579401003042 11523844

[ejp4702-bib-0005] Brockie, T. N. , Dana‐Sacco, G. , Wallen, G. R. , Wilcox, H. C. , & Campbell, J. C. (2015). The relationship of adverse childhood experiences to PTSD, depression, poly‐drug use and suicide attempt in reservation‐based native american adolescents and young adults. American Journal of Community Psychology, 55(3), 411–421. 10.1007/s10464-015-9721-3 25893815

[ejp4702-bib-0006] Burke Harris, N. , & Renschler, T. (2015). Center for Youth Wellness ACE‐Questionnaire (CYW ACE‐Q Child, Teen, Teen SR).

[ejp4702-bib-0007] Carskadon, M. A. , & Acebo, C. (1993). A self‐administered rating scale for pubertal development. Journal of Adolescent Health, 14(3), 190–195. 10.1016/1054-139x(93)90004-9 8323929

[ejp4702-bib-0008] Cassiers, L. L. M. , Sabbe, B. G. C. , Schmaal, L. , Veltman, D. J. , Penninx, B. , & Van Den Eede, F. (2018). Structural and functional brain abnormalities associated with exposure to different childhood trauma subtypes: A systematic review of neuroimaging findings. Frontiers in Psychiatry, 9, 329. 10.3389/fpsyt.2018.00329 30123142 PMC6086138

[ejp4702-bib-0009] Cisler, J. M. , Privratsky, A. , Smitherman, S. , Herringa, R. J. , & Kilts, C. D. (2018). Large‐scale brain organization during facial emotion processing as a function of early life trauma among adolescent girls. NeuroImage Clinical, 17, 778–785. 10.1016/j.nicl.2017.12.001 29527485 PMC5842665

[ejp4702-bib-0010] Cornelissen, L. , Donado, C. , Kim, J. , Chiel, L. , Zurakowski, D. , Logan, D. E. , Meier, P. , Sethna, N. F. , Blankenburg, M. , Zernikow, B. , Sundel, R. P. , & Berde, C. B. (2014). Pain hypersensitivity in juvenile idiopathic arthritis: A quantitative sensory testing study. Pediatric Rheumatology, 12(1), 39. 10.1186/1546-0096-12-39 25249820 PMC4171552

[ejp4702-bib-0011] Cox, R. W. (1996). AFNI: Software for analysis and visualization of functional magnetic resonance neuroimages. Computers and Biomedical Research, 29(3), 162–173. 10.1006/cbmr.1996.0014 8812068

[ejp4702-bib-0012] Foa, E. B. , Johnson, K. M. , Feeny, N. C. , & Treadwell, K. R. (2001). The child PTSD symptom scale: A preliminary examination of its psychometric properties. Journal of Clinical Child Psychology, 30(3), 376–384. 10.1207/S15374424JCCP3003_9 11501254

[ejp4702-bib-0013] Fonov, V. , Evans, A. C. , Botteron, K. , Almli, C. R. , McKinstry, R. C. , & Collins, D. L. (2011). Unbiased average age‐appropriate atlases for pediatric studies. NeuroImage, 54(1), 313–327. 10.1016/j.neuroimage.2010.07.033 20656036 PMC2962759

[ejp4702-bib-0014] Gracia‐Tabuenca, Z. , Moreno, M. B. , Barrios, F. A. , & Alcauter, S. (2021). Development of the brain functional connectome follows puberty‐dependent nonlinear trajectories. NeuroImage, 229, 117769. 10.1016/j.neuroimage.2021.117769 33482398

[ejp4702-bib-0015] Graf, G. H. , Biroli, P. , & Belsky, D. W. (2021). Critical periods in child development and the transition to adulthood. JAMA Network Open, 4(1), e2033359. 10.1001/jamanetworkopen.2020.33359 33410874

[ejp4702-bib-0016] Granovsky, Y. , & Yarnitsky, D. (2013). Personalized pain medicine: The clinical value of psychophysical assessment of pain modulation profile. Rambam Maimonides Medical Journal, 4(4), e0024. 10.5041/rmmj.10131 24228167 PMC3820297

[ejp4702-bib-0017] Greene, J. W. , Walker, L. S. , Hickson, G. , & Thompson, J. (1985). Stressful life events and somatic complaints in adolescents. Pediatrics, 75(1), 19–22. 10.1016/S0002-7138(09)61122-5 3966041

[ejp4702-bib-0018] Groenewald, C. B. , Murray, C. B. , & Palermo, T. M. (2020). Adverse childhood experiences and chronic pain among children and adolescents in the United States. Pain Reports, 5(5), e839. 10.1097/pr9.0000000000000839 32903388 PMC7431222

[ejp4702-bib-0019] Hagander, L. G. , Midani, H. A. , Kuskowski, M. A. , & Parry, G. J. (2000). Quantitative sensory testing: Effect of site and skin temperature on thermal thresholds. Clinical Neurophysiology, 111(1), 17–22. 10.1016/s1388-2457(99)00192-3 10656506

[ejp4702-bib-0020] Hayes, A. F. (2012). *PROCESS: A versatile computational tool for observed variable mediation, moderation, and conditional process modeling* [White paper]. Retrieved from https://www.afhayes.com/public/process2012.pdf

[ejp4702-bib-0021] Herzog, J. I. , & Schmahl, C. (2018). Adverse childhood experiences and the consequences on neurobiological, psychosocial, and somatic conditions across the lifespan. Frontiers in Psychiatry, 9, 420. 10.3389/fpsyt.2018.00420 30233435 PMC6131660

[ejp4702-bib-0022] Holley, A. L. , Wilson, A. C. , Noel, M. , & Palermo, T. M. (2016). Post‐traumatic stress symptoms in children and adolescents with chronic pain: A topical review of the literature and a proposed framework for future research. European Journal of Pain, 20(9), 1371–1383. 10.1002/ejp.879 27275585 PMC5912261

[ejp4702-bib-0023] IBM Corp . (2021). IBM SPSS statistics for Macintosh (version 28.0) [Computer software]. IBM Corporation.

[ejp4702-bib-0024] Jacob, E. , Chan, V. W. , Hodge, C. , Zeltzer, L. , Zurakowski, D. , & Sethna, N. F. (2015). Sensory and thermal quantitative testing in children with sickle cell disease. Journal of Pediatric Hematology/Oncology, 37(3), 185–189. 10.1097/MPH.0000000000000214 25014619 PMC6589156

[ejp4702-bib-0025] Janssen, J. , Abou‐Assaly, E. , Rasic, N. , Noel, M. , & Miller, J. V. (2022). Trauma and pain sensitization in youth with chronic pain. PAIN Reports, 7(2), e992. 10.1097/PR9.0000000000000992 35317187 PMC8929520

[ejp4702-bib-0026] Jenkinson, M. , Beckmann, C. F. , Behrens, T. E. J. , Woolrich, M. W. , & Smith, S. M. (2012). FSL. NeuroImage, 62(2), 782–790. 10.1016/j.neuroimage.2011.09.015 21979382

[ejp4702-bib-0027] Kerker, B. D. , Zhang, J. , Nadeem, E. , Stein, R. E. , Hurlburt, M. S. , Heneghan, A. , Landsverk, J. , & McCue Horwitz, S. (2015). Adverse childhood experiences and mental health, chronic medical conditions, and development in young children. Academic Pediatrics, 15(5), 510–517. 10.1016/j.acap.2015.05.005 26183001 PMC4562867

[ejp4702-bib-0028] Khera, T. , & Rangasamy, V. (2021). Cognition and pain: A review. Frontiers in Psychology, 12, 673962. 10.3389/fpsyg.2021.673962 34093370 PMC8175647

[ejp4702-bib-0029] King, S. , Chambers, C. T. , Huguet, A. , MacNevin, R. C. , McGrath, P. J. , Parker, L. , & MacDonald, A. J. (2011). The epidemiology of chronic pain in children and adolescents revisited: A systematic review. Pain, 152(12), 2729–2738. 10.1016/j.pain.2011.07.016 22078064

[ejp4702-bib-0030] Koenis, M. M. G. , Brouwer, R. M. , Swagerman, S. C. , van Soelen, I. L. C. , Boomsma, D. I. , & Hulshoff Pol, H. E. (2018). Association between structural brain network efficiency and intelligence increases during adolescence. Human Brain Mapping, 39(2), 822–836. 10.1002/hbm.23885 29139172 PMC6866576

[ejp4702-bib-0031] Korem, N. , Duek, O. , Ben‐Zion, Z. , Kaczkurkin, A. N. , Lissek, S. , Orederu, T. , Schiller, D. , Harpaz‐Rotem, I. , & Levy, I. (2022). Emotional numbing in PTSD is associated with lower amygdala reactivity to pain. Neuropsychopharmacology, 47(11), 1913–1921. 10.1038/s41386-022-01405-2 35945274 PMC9485255

[ejp4702-bib-0032] Larsen, B. , & Luna, B. (2018). Adolescence as a neurobiological critical period for the development of higher‐order cognition. Neuroscience & Biobehavioral Reviews, 94, 179–195. 10.1016/j.neubiorev.2018.09.005 30201220 PMC6526538

[ejp4702-bib-0033] Latora, V. , & Marchiori, M. (2001). Efficient behavior of small‐world networks. Physical Review Letters, 87(19), 198701. 10.1103/PhysRevLett.87.198701 11690461

[ejp4702-bib-0034] Lei, D. , Li, K. , Li, L. , Chen, F. , Huang, X. , Lui, S. , Li, J. , Bi, F. , & Gong, Q. (2015). Disrupted functional brain connectome in patients with posttraumatic stress disorder. Radiology, 278(3), 818–827. 10.1148/radiol.15141700 25848901

[ejp4702-bib-0035] Lenoir, D. , Cagnie, B. , Verhelst, H. , & De Pauw, R. (2021). Graph measure based connectivity in chronic pain patients: A systematic review. Pain Physician, 24(7), E1037–E1058. 34704714

[ejp4702-bib-0036] Lowry, E. , McInerney, A. , Schmitz, N. , & Deschênes, S. S. (2022). Adverse childhood experiences and cognitive function in adulthood: Examining the roles of depressive symptoms and inflammation in a prospective cohort study. Social Psychiatry and Psychiatric Epidemiology, 57(12), 2367–2377. 10.1007/s00127-022-02315-w 35753000 PMC9244111

[ejp4702-bib-0037] Malmström, E.‐M. , Stjerna, J. , Högestätt, E. D. , & Westergren, H. (2015). Quantitative sensory testing of temperature thresholds: Possible biomarkers for persistent pain? Journal of Rehabilitation Medicine, 48(1), 43–47. 10.2340/16501977-2024 26450179

[ejp4702-bib-0038] Mateos‐Aparicio, P. , & Rodríguez‐Moreno, A. (2019). The impact of studying brain plasticity. Frontiers in Cellular Neuroscience, 13, 66. 10.3389/fncel.2019.00066 30873009 PMC6400842

[ejp4702-bib-0039] Müller, H. (2000). Neuroplasticity and chronic pain. Anästhesiologie, Intensivmedizin, Notfallmedizin, Schmerztherapie, 35(5), 274–284. 10.1055/s-2000-352 10858836

[ejp4702-bib-0040] Nelson, C. A. , Scott, R. D. , Bhutta, Z. A. , Harris, N. B. , Danese, A. , & Samara, M. (2020). Adversity in childhood is linked to mental and physical health throughout life. BMJ, 371, m3048. 10.1136/bmj.m3048 33115717 PMC7592151

[ejp4702-bib-0041] Nelson, S. M. , Cunningham, N. R. , & Kashikar‐Zuck, S. (2017). A conceptual framework for understanding the role of adverse childhood experiences in pediatric chronic pain. Clinical Journal of Pain, 33(3), 264–270. 10.1097/ajp.0000000000000397 27275737 PMC5143226

[ejp4702-bib-0042] Neziri, A. Y. , Scaramozzino, P. , Andersen, O. K. , Dickenson, A. H. , Arendt‐Nielsen, L. , & Curatolo, M. (2011). Reference values of mechanical and thermal pain tests in a pain‐free population. European Journal of Pain, 15(4), 376–383. 10.1016/j.ejpain.2010.08.011 20932788

[ejp4702-bib-0043] Noel, M. , Wilson, A. C. , Holley, A. L. , Durkin, L. , Patton, M. , & Palermo, T. M. (2016). Posttraumatic stress disorder symptoms in youth with vs without chronic pain. Pain, 157(10), 2277–2284. 10.1097/j.pain.0000000000000642 27276275 PMC5028262

[ejp4702-bib-0044] Ohashi, K. , Anderson, C. M. , Bolger, E. A. , Khan, A. , McGreenery, C. E. , & Teicher, M. H. (2017). Childhood maltreatment is associated with alteration in global network fibre‐tract architecture independent of history of depression and anxiety. NeuroImage, 150, 50–59. 10.1016/j.neuroimage.2017.02.037 28213111 PMC5386807

[ejp4702-bib-0045] Power, J. D. , Mitra, A. , Laumann, T. O. , Snyder, A. Z. , Schlaggar, B. L. , & Petersen, S. E. (2014). Methods to detect, characterize, and remove motion artifact in resting state fMRI. NeuroImage, 84, 320–341. 10.1016/j.neuroimage.2013.08.048 23994314 PMC3849338

[ejp4702-bib-0046] Raffaeli, W. , & Arnaudo, E. (2017). Pain as a disease: An overview. Journal of Pain Research, 10, 2003–2008. 10.2147/JPR.S138864 28860855 PMC5573040

[ejp4702-bib-0047] Reckziegel, D. , Vachon‐Presseau, E. , Petre, B. , Schnitzer, T. J. , Baliki, M. N. , & Apkarian, A. V. (2019). Deconstructing biomarkers for chronic pain: Context‐ and hypothesis‐dependent biomarker types in relation to chronic pain. Pain, 160(Suppl 1), S37–S48. 10.1097/j.pain.0000000000001529 31008848 PMC6478400

[ejp4702-bib-0048] Salberg, S. , Doshen, A. , Yamakawa, G. , Vinall Miller, J. , Noel, M. , Henderson, L. , & Mychasiuk, R. (2022). The waiting game: Investigating the neurobiological transition from acute to persistent pain in adolescent rats. Cerebral Cortex, bhac511, 6382–6393. 10.1093/cercor/bhac511 PMC10183733 36610738

[ejp4702-bib-0049] Satterthwaite, T. D. , Elliott, M. A. , Gerraty, R. T. , Ruparel, K. , Loughead, J. , Calkins, M. E. , Eickhoff, S. B. , Hakonarson, H. , Gur, R. C. , Gur, R. E. , & Wolf, D. H. (2013). An improved framework for confound regression and filtering for control of motion artifact in the preprocessing of resting‐state functional connectivity data. NeuroImage, 64, 240–256. 10.1016/j.neuroimage.2012.08.052 22926292 PMC3811142

[ejp4702-bib-0050] Schulte, T. , Hong, J. Y. , Sullivan, E. V. , Pfefferbaum, A. , Baker, F. C. , Chu, W. , Prouty, D. , Kwon, D. , Meloy, M. J. , Brumback, T. , Tapert, S. F. , Colrain, I. M. , & Müller‐Oehring, E. M. (2020). Effects of age, sex, and puberty on neural efficiency of cognitive and motor control in adolescents. Brain Imaging and Behavior, 14(4), 1089–1107. 10.1007/s11682-019-00075-x 30903550 PMC6756998

[ejp4702-bib-0051] Seitzman, B. A. , Snyder, A. Z. , Leuthardt, E. C. , & Shimony, J. S. (2019). The state of resting state networks. Topics in Magnetic Resonance Imaging, 28(4), 189–196. 10.1097/rmr.0000000000000214 31385898 PMC6686880

[ejp4702-bib-0052] Sheynin, J. , Duval, E. R. , Lokshina, Y. , Scott, J. C. , Angstadt, M. , Kessler, D. , Zhang, L. , Gur, R. E. , Gur, R. C. , & Liberzon, I. (2020). Altered resting‐state functional connectivity in adolescents is associated with PTSD symptoms and trauma exposure. NeuroImage: Clinical, 26, 102215. 10.1016/j.nicl.2020.102215 32339825 PMC7184176

[ejp4702-bib-0053] Stanford, E. A. , Chambers, C. T. , Biesanz, J. C. , & Chen, E. (2008). The frequency, trajectories and predictors of adolescent recurrent pain: A population‐based approach. Pain, 138(1), 11–21. 10.1016/j.pain.2007.10.032 18093737

[ejp4702-bib-0054] Suo, X. , Lei, D. , Li, K. , Chen, F. , Li, F. , Li, L. , Huang, X. , Lui, S. , Li, L. , Kemp, G. J. , & Gong, Q. (2015). Disrupted brain network topology in pediatric posttraumatic stress disorder: A resting‐state fMRI study. Human Brain Mapping, 36(9), 3677–3686. 10.1002/hbm.22871 26096541 PMC6869652

[ejp4702-bib-0055] Tidmarsh, L. V. , Harrison, R. , Ravindran, D. , Matthews, S. L. , & Finlay, K. A. (2022). The influence of adverse childhood experiences in pain management: Mechanisms, processes, and trauma‐informed care. Frontiers in Pain Research (Lausanne), 3, 923866. 10.3389/fpain.2022.923866 PMC9226323 35756908

[ejp4702-bib-0056] Tsze, D. S. , von Baeyer, C. L. , Pahalyants, V. , & Dayan, P. S. (2018). Validity and reliability of the verbal numerical rating scale for children aged 4 to 17 years with acute pain. Annals of Emergency Medicine, 71(6), 691–702. 10.1016/j.annemergmed.2017.09.009 29107409 PMC5920794

[ejp4702-bib-0057] Tzourio‐Mazoyer, N. , Landeau, B. , Papathanassiou, D. , Crivello, F. , Etard, O. , Delcroix, N. , Mazoyer, B. , & Joliot, M. (2002). Automated anatomical labeling of activations in SPM using a macroscopic anatomical parcellation of the MNI MRI single‐subject brain. NeuroImage, 15(1), 273–289. 10.1006/nimg.2001.0978 11771995

[ejp4702-bib-0058] Varni, J. W. , Magnus, B. , Stucky, B. D. , Liu, Y. , Quinn, H. , Thissen, D. , Gross, H. E. , Huang, I. C. , & DeWalt, D. A. (2014). Psychometric properties of the PROMIS (R) pediatric scales: Precision, stability, and comparison of different scoring and administration options. Quality of Life Research, 23, 1233–1243. 24085345 10.1007/s11136-013-0544-0 PMC4312615

[ejp4702-bib-0059] Vinall, J. , Pavlova, M. , Asmundson, G. J. , Rasic, N. , & Noel, M. (2016). Mental health comorbidities in pediatric chronic pain: A narrative review of epidemiology, models, neurobiological mechanisms and treatment. Children (Basel), 3(4), 40. 10.3390/children3040040 27918444 PMC5184815

[ejp4702-bib-0060] Wang, J. , Wang, X. , Xia, M. , Liao, X. , Evans, A. , & He, Y. (2015). GRETNA: A graph theoretical network analysis toolbox for imaging connectomics. Frontiers in Human Neuroscience, 9, 386. 10.3389/fnhum.2015.00386 26175682 PMC4485071

[ejp4702-bib-0061] Wang, R. , Liu, M. , Cheng, X. , Wu, Y. , Hildebrandt, A. , & Zhou, C. (2021). Segregation, integration, and balance of large‐scale resting brain networks configure different cognitive abilities. Proceedings of the National Academy of Sciences, 118(23), e2022288118. 10.1073/pnas.2022288118 PMC8201916 34074762

[ejp4702-bib-0062] Weaver, K. R. , Griffioen, M. A. , Klinedinst, N. J. , Galik, E. , Duarte, A. C. , Colloca, L. , Resnick, B. , Dorsey, S. G. , & Renn, C. L. (2021). Quantitative sensory testing across chronic pain conditions and use in special populations. Frontiers in Pain Research (Lausanne), 2, 779068. 10.3389/fpain.2021.779068 PMC8915716 35295425

[ejp4702-bib-0063] Wheelock, M. D. , Rangaprakash, D. , Harnett, N. G. , Wood, K. H. , Orem, T. R. , Mrug, S. , Granger, D. A. , Deshpande, G. , & Knight, D. C. (2018). Psychosocial stress reactivity is associated with decreased whole‐brain network efficiency and increased amygdala centrality. Behavioral Neuroscience, 132(6), 561–572. 10.1037/bne0000276 30359065 PMC6242743

[ejp4702-bib-0064] Wig, G. S. (2017). Segregated systems of human brain networks. Trends in Cognitive Sciences, 21(12), 981–996. 10.1016/j.tics.2017.09.006 29100737

[ejp4702-bib-0065] Wolff, N. , Rubia, K. , Knopf, H. , Hölling, H. , Martini, J. , Ehrlich, S. , & Roessner, V. (2016). Reduced pain perception in children and adolescents with ADHD is normalized by methylphenidate. Child and Adolescent Psychiatry and Mental Health, 10, 24. 10.1186/s13034-016-0112-9 27453723 PMC4957360

[ejp4702-bib-0066] Wozniak, J. R. , Mueller, B. A. , Mattson, S. N. , Coles, C. D. , Kable, J. A. , Jones, K. L. , Boys, C. J. , Lim, K. O. , Riley, E. P. , & Sowell, E. R. (2017). Functional connectivity abnormalities and associated cognitive deficits in fetal alcohol spectrum disorders (FASD). Brain Imaging and Behavior, 11(5), 1432–1445. 10.1007/s11682-016-9624-4 27734306 PMC5389933

[ejp4702-bib-0067] Xia, M. , Wang, J. , & He, Y. (2013). BrainNet viewer: A network visualization tool for human brain connectomics. PLoS One, 8(7), e68910. 10.1371/journal.pone.0068910 23861951 PMC3701683

[ejp4702-bib-0068] Xu, J. , Chen, F. , Lei, D. , Zhan, W. , Sun, X. , Suo, X. , Peng, Z. , Wang, T. , Zhang, J. , & Gong, Q. (2018). Disrupted functional network topology in children and adolescents with post‐traumatic stress disorder. Frontiers in Neuroscience, 12, 709. 10.3389/fnins.2018.00709 30356635 PMC6189287

[ejp4702-bib-0069] Zheng, W. , Woo, C. W. , Yao, Z. , Goldstein, P. , Atlas, L. Y. , Roy, M. , Schmidt, L. , Krishnan, A. , Jepma, M. , Hu, B. , & Wager, T. D. (2020). Pain‐evoked reorganization in functional brain networks. Cerebral Cortex, 30(5), 2804–2822. 10.1093/cercor/bhz276 31813959 PMC7197093

