# Supplementary material for: Adverse childhood experiences, brain efficiency, and the development of pain symptoms in youth
Source: Eur J Pain. 2024 Jul 16;29(1):e4702. doi: 10.1002/ejp.4702 (PMC11609899; doi:10.1002/ejp.4702)
Supplement: Supplementary file 1 — Tables S1–S2. [file EJP-29-0-s001.docx]

**Supplementary material**

**Table S1 Main Themes of Traumatic Events Identified by Youth on the CPSS-V**

| **Types of Traumatic Events** | **Baseline (*n* = 44),**  **N (%)** | **Follow-up (*n* = 42),**  **N (%)** |
| --- | --- | --- |
| Concerns for the mental health of a household member or peer, including suicide ideation and addiction | 6 (13.64) | 7 (16.67) |
| Abuse | 5 (11.36) | 4 (9.52) |
| Physical abuse | 1 (2.27) | 0 (0.00) |
| Emotional abuse | 3 (6.82) | 1 (2.38) |
| Sexual abuse | 0 (0.00) | 1 (2.38) |
| Abuse from a romantic partner | 1 (2.27) | 2 (4.76) |
| Death/loss of a family member, close friend, or pet | 4 (9.10) | 4 (9.52) |
| Felt unloved, unsupported, or unwanted by others, including bullying | 4 (9.10) | 3 (7.14) |
| Concerns for the physical health of others | 4 (9.10) | 3 (7.14) |
| Concerns for personal health and well-being | 3 (6.82) | 1 (2.38) |
| Household conflict (e.g., caregivers fighting), including parental separation/divorce | 2 (4.55) | 4 (9.52) |
| General neglect from caregivers | 2 (4.55) | 1 (2.38) |
| Sexual assault | 1 (2.27) | 1 (2.38) |
| Raised in foster care | 1 (2.27) | 0 (0.00) |
| Experienced a serious accident (e.g., motor vehicle) | 1 (2.27) | 0 (0.00) |
| Interpersonal conflict with others | 0 (0.00) | 2 (4.76) |
| Non-specified | 7 (15.91) | 9 (21.43) |
| No trauma | 4 (9.10) | 3 (7.14) |

**Table S2 Trauma and Brain Efficiency**

|  | **Global Efficiency** | | | **Local Efficiency** | | |
| --- | --- | --- | --- | --- | --- | --- |
| **Parameter** | ***β*** | **95% CI** | ***P*-value** | ***β*** | **95% CI** | ***P*-value** |
| Age | 0.09 | [-0.20, 0.21] | 0.52 | 0.03 | [-0.23, 0.28] | 0.85 |
| Gender | 0.12 | [-0.19, 0.37] | 0.35 | 0.12 | [-0.09, 0.33] | 0.28 |
| Puberty | -0.21 | [-0.52, 0.11] | 0.20 | -0.18 | [-0.48, 0.11] | 0.23 |
| Anxiety | -0.06 | [-0.42, 0.30] | 0.75 | -0.06 | [-0.40, 0.28] | 0.72 |
| Depression | 0.15 | [-0.07, 0.37] | 0.17 | 0.17 | [-0.07, 0.42] | 0.17 |
| PTSS | 0.11 | [-0.17, 0.39] | 0.43 | 0.15 | [-0.16, 0.46] | 0.34 |
| ACEs | -0.23 | [-0.44, -0.02] | **0.03*** | -0.26 | [-0.46, -0.07] | **0.01*** |

*ACEs = number of exposures to adverse childhood experiences; PTSS = mean posttraumatic stress symptom score*

**P ≤ 0.05.*
